# Diaphragmatic Rupture in the Postpartum Period

**DOI:** 10.7759/cureus.75807

**Published:** 2024-12-16

**Authors:** Ibtissam Nabih, Khalid Khaleq, Khalid Elhattabi

**Affiliations:** 1 Anesthesia and Critical Care, Ibn Rochd University Hospital/Hassan II University, Casablanca, MAR; 2 General Surgery, Ibn Rochd University Hospital/Hassan II University, Casablanca, MAR

**Keywords:** diaphragmatic defect repair, diaphragmatic rupture, labor complications, postpartum respiratory symptoms, unsupervised delivery

## Abstract

Diaphragmatic rupture during labor is an exceptionally rare condition, with a limited number of cases reported in the literature. A recent review underscores the rarity of this complication and emphasizes the associated challenges in diagnosis and management. This case report presents a postpartum diaphragmatic rupture, focusing on the diagnostic and therapeutic challenges it poses, particularly in the context of unsupervised deliveries.

We report the case of a 32-year-old woman, with no significant medical history, who presented with intractable vomiting, and progressively worsening exertional dyspnea 30 days after a normal vaginal delivery. A chest X-ray revealed an air-filled space above the diaphragm, and an abdominal CT scan confirmed a diaphragmatic rupture with herniation of the stomach into the thoracic cavity, causing upward shifting of the cardiac chambers. The patient underwent emergency surgery via a midline supra-umbilical laparotomy. The herniated stomach was successfully repositioned into the abdominal cavity, the diaphragmatic defect was repaired, and an anti-reflux posterior hemivalve was created. Postoperative recovery was smooth, with significant improvement in clinical symptoms.

This case highlights the importance of considering diaphragmatic rupture in postpartum women presenting with unexplained respiratory symptoms, particularly in settings where labor is not medically supervised. Rapid surgical intervention is crucial to prevent life-threatening complications and improve maternal outcomes.

## Introduction

Diaphragmatic rupture during labor is an exceptionally rare condition, with a limited number of cases reported in the literature [1,2]. The diagnosis must be made before potentially fatal complications set in. Radiological investigations can confirm the diagnosis. Treatment is by emergency surgery, which is the only way to improve the prognosis of this condition [3]. We report a new case of postpartum diaphragmatic rupture, diagnosed and managed in the P35 emergency surgical unit of the Ibn Rochd University Hospital, Casablanca.

## Case presentation

A 32-year-old patient, with no significant medical history, was hospitalized in an emergency setting 30 days after a vaginal delivery. She presented with intractable vomiting and progressively worsening exertional dyspnea.

**Figure 1:**
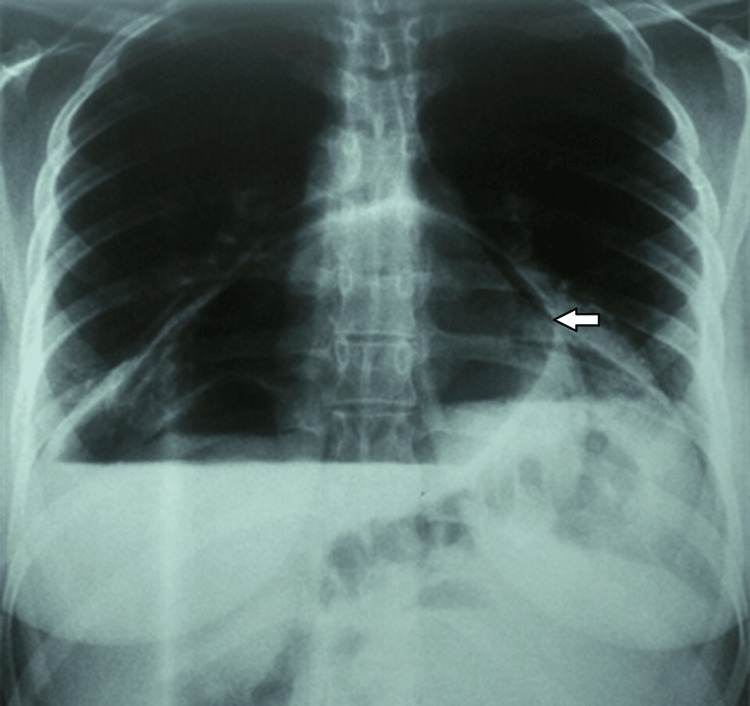
Aeriform image above the diaphragm projecting behind the cardiac opacity on chest X-ray

The chest X-ray revealed an air-filled space above the diaphragm, projected behind the cardiac shadow (Figure 1).

**Figure 2:**
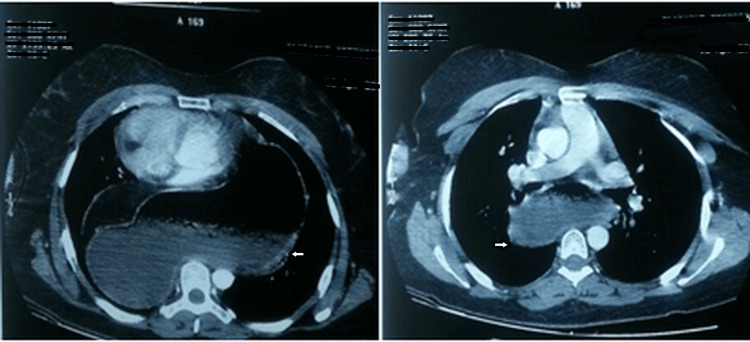
Diaphragmatic hernia and intrathoracic gastric fluid distension on thoraco-abdominal CT scan

An abdominal CT scan showed a diaphragmatic rupture with intrathoracic herniation of the stomach, causing an upward displacement of the cardiac chambers without small or large bowel obstruction (Figure 2).

The surgical Intervention was performed via a midline supra-umbilical laparotomy. Exploration revealed a herniated stomach in the thorax without peritoneal effusion. The treatment consisted of repositioning the viable stomach into the abdominal cavity, repairing the diaphragmatic defect, and a posterior partial fundoplication was performed to prevent reflux. The patient was then admitted to the surgical intensive care unit P33. Postoperative recovery was smooth, with significant improvement in clinical symptoms.

## Discussion

Postpartum diaphragmatic rupture is a rare but potentially fatal condition requiring immediate surgical intervention to prevent serious complications for the mother. In regions where non-medicalized deliveries are common, such as in parts of Morocco, the risk of complications like diaphragmatic rupture may be amplified.

Diaphragmatic hernias can be congenital or acquired, often secondary to thoraco-abdominal trauma. However, the physiological changes of pregnancy, marked by increased intra-abdominal pressure, play a crucial role in either revealing or exacerbating a pre-existing diaphragmatic defect. As reported in several studies [2,3], the increased intra-abdominal pressure during pregnancy and labor can provoke or worsen a diaphragmatic hernia, which may then manifest with severe respiratory symptoms and cardiopulmonary distress [1].

In the literature, the clinical presentation of postpartum diaphragmatic rupture often includes symptoms such as epigastric pain, intractable vomiting, and progressive dyspnea, as was the case in our patient. In a study by Hill and Heller, a patient presented with intense back pain and severe dyspnea a few hours after labor, which ultimately led to the diagnosis of diaphragmatic rupture after a chest X-ray revealed herniation of abdominal organs into the thorax [4]. Similarly, our patient also presented with aggravated exertional dyspnea and intractable vomiting, with radiographic signs of a diaphragmatic hernia.

Another case reported by Fleyfel et al. describes a patient with a history of abdominal surgery who, after several weeks of vomiting and abdominal pain, was diagnosed with a diaphragmatic hernia following a chest X-ray and abdominal CT scan [3]. In our case, the absence of surgical history did not prevent the development of a diaphragmatic rupture, suggesting that other factors, such as increased intra-abdominal pressure during labor, may play a determining role.

Management of diaphragmatic rupture involves immediate surgical intervention. Surgical exploration, generally performed via laparotomy, allows for the reduction of herniated organs into the abdominal cavity and the repair of the diaphragmatic defect. A laparoscopic approach has also been reported in some uncomplicated cases, although open surgery is often necessary in emergency situations with complications. The literature supports the idea that rapid surgical management is essential to reduce maternal mortality, especially in contexts where complications may go unnoticed due to the lack of structured medical follow-up [1,3,5]. In the presented case, the repositioning of the stomach into the abdominal cavity and the creation of an anti-reflux hemivalve resulted in a favorable outcome without major postoperative complications.

In deliveries occurring without adequate medical supervision, the absence of qualified personnel can delay surgical intervention, thereby increasing the risk of severe complications. A multidisciplinary approach encompassing thoracic surgeons, obstetricians, anesthetists, and radiologists is often essential for managing such complex cases, but this level of care may not be available in non-medicalized settings [1,6,7].

The prognosis of diaphragmatic ruptures largely depends on the speed of diagnosis and surgical intervention [7]. Case studies show that delays in recognizing this condition can lead to severe complications, such as organ necrosis or cardiopulmonary collapse, thereby increasing maternal mortality [8,9]. Rapid management, as described in this case, is essential to improve prognosis [10].

Our case is particularly significant in the Moroccan context, where non-medicalized deliveries remain frequent in some rural areas. This context exposes patients to an increased risk of severe complications due to certain abdominal expression maneuvers still practiced in some non-medicalized deliveries. Additionally, it illustrates the importance of clinical vigilance, especially in recognizing non-specific signs, as well as the central role of medical imaging in diagnosis. Finally, it highlights the vital nature of rapid surgical intervention to prevent potentially fatal complications and ensure a favorable outcome for patients.

## Conclusions

Diaphragmatic rupture during pregnancy and labor is an uncommon condition but, especially in the case of postpartum diagnosis, may be life-threatening. The abdominal viscera may be incarcerated in the thoracic cavity; as a result, cardiopulmonary compromise may occur with serious complications for both the mother and fetus. This further attests to the necessity for heightened clinical suspicion and complete diagnostic workup in pregnant or postpartum patients presenting with unexplained respiratory or abdominal complaints. Our case adds to the literature and illustrates the importance of early recognition and immediate surgical management of patients for a favorable outcome.
